# High-grade non-muscle invasive urothelial carcinoma in dogs and humans share specific expression of integrin α5β1

**DOI:** 10.3389/fonc.2025.1590073

**Published:** 2025-05-28

**Authors:** Roberta Lucianò, Maurizio Colecchia, Francesca Sanvito, Irene Locatelli, Chiara Venegoni, Alessia Di Coste, Davide Danilo Zani, Angelica Stranieri, Chiara Giudice, Antonella Rigillo, Matteo Gambini, Francesco Montorsi, Andrea Salonia, Marco Moschini, Massimo Alfano

**Affiliations:** ^1^ Department of Pathology, Istituto di Ricovero e Cura a Caratterle Scientifico (IRCCS) San Raffaele Scientific Institute, Milan, Italy; ^2^ Università Vita-Salute San Raffaele, Milan, Italy; ^3^ Division of Experimental Oncology/Unit of Urology, Urological Research Institute (URI), Istituto di Ricovero e Cura a Caratterle Scientifico (IRCCS) San Raffaele Scientific Institute, Milan, Italy; ^4^ Department of Veterinary Medicine and Animal Science, University of Milan, Lodi, Italy; ^5^ I-Vet Diagnostica Veterinaria, Brescia, Italy

**Keywords:** urothelial cancer, human, dog, integrin, marker, bladder

## Abstract

**Objective:**

Urothelial carcinoma (UC) accounts for more than 90% of all bladder cancers both in humans and dogs. Human and canine UC share many genetic mutations and tumor markers and clinical and therapeutic interventions. The unmet clinical needs are similar such as the early detection and treatment of the high-grade residual disease responsible for tumor recurrence and progression. The aim of this study was to investigate the expression of the α5β1 integrin and its specificity in high-grade UC in humans and dogs, a marker recently reported in the human bladder *in situ* carcinoma and murine model of orthotopic bladder cancer.

**Methods:**

Expression of integrin α5β1 was established by immunohistochemistry in 67 human bladder samples [four non-tumor tissues, 10 low-grade, 10 intermediate-grade, and 43 high-grade non-muscle invasive bladder cancer (NMIBC)] and 12 canine bladder tumor specimens.

**Results:**

The α5β1 integrin was not expressed by urothelial cells in the conditions of inflammatory cystitis, actinic cystitis, benign hyperplasia, and low/intermediate grade NMIBC; it was identified as a specific marker expressed only by the malignant cells in the urothelium in 81% of human and all canine high-grade NMIBC.

**Conclusions:**

The expression of α5β1 integrin is a specific marker of high-grade UC located in the urothelium of humans and dogs and might be tested for targeted delivery of contrast agents or drugs. Given the close similarity between high-grade UC in humans and dogs, basic research in the two species and comparative data analysis could strengthen the prospects for rapid development of an improved clinical strategy for the identification and treatment of the small neoplastic lesions responsible for residual high-grade in both species.

## Introduction

One of the unmet clinical needs in the management of urothelial carcinoma (UC) is the identification and treatment of the residual disease after optimal treatment, responsible for disease relapse and progression. The bladder urothelium is uniformly exposed to the carcinogens with several areas of normal tissue affected by mutated clones present at the same time, named “field cancerization effect.” For patients with high-risk non-muscle-invasive bladder cancer (NMIBC), the “field cancerization effect” is associated with the development and recurrence of bladder cancer and may be associated with treatment outcomes (1), and in the preclinical model, the topographic modification of the extracellular matrix induced by radiotherapy sustains early onset of tumor growth induced by nitrosamine (2).

Most human and canine tumors share molecular genetics and clinical similarities (3). UC represents approximately 2% of all cancers, both in human (4) and dogs (5). The onset of UC is common in several canine breeds, with the highest odds ratio in the Scottish terrier (6) accounting for 11.7% of this breed (5).

In humans, 75% of UC are NMIBC at the first diagnosis (7), while in dogs, more than 90% are MIBC (81% papillary and 14% non-papillary UC) (5, 8) and high-grade in 76% (6, 8). For canine UC partial cystectomy and medical therapy are available. Indeed, disease progression due to recurrence occurs in the majority of animals treated with partial cystectomy (66%–100%) (8, 9), and the median progression-free survival after adjuvant therapy is 83–101 days (9, 10) and 119 days for medical therapy with intravesical instillation of Mitomycin C (11). Likewise, in humans, recurrence of bladder cancer at 2 and 5 years occurs in 36% and 50% of patients with high-grade NMIBC treated with *Bacillus* Calmette–Guerin (BCG) (12), and 10%–30% may progress at 5 years (13, 14).

Furthermore, human and canine UC share the same tumoral targets: among others, epithelial growth factor receptor (EGFR) that is overexpressed in 75% of high-grade human UC and 73% of canine muscle-invasive high-grade UC and cyclooxygenase-2 (Cox-2) that is overexpressed in 80% of both human and canine muscle-invasive high-grade UC (5). Likewise, dogs and humans share similar gut and urinary microbiomes (15–18), making dogs a more reliable model than rodents for studying UC.

We have recently characterized a murine model of orthotopic bladder cancer whose neoplastic cells express the integrin α5β1, an integrin that is also expressed in 81% of human high-grade NMIBC (19, 20). In the preclinical murine model, we also demonstrated that targeting the integrin α5β1 with engineered gold nanorods allows the detection and treatment of cancer lesions <1 mm (19, 20). Apart from developing the orthotopic bladder tumor, the preclinical model based on the intravesical instillation of MB49 bladder cancer cells does not show other signs of disease, for example, inflammatory reactions, as instead present in clinical specimens.

In this study, we deepen the integrin α5β1 expression pattern in the urothelium, by providing detailed pathological and therapeutical data of a large cohort of human high-grade NMIBC, low- and intermediate-grade NMIBC and non-oncological clinical specimens and included cases of canine spontaneous high-grade UC. We aimed to assess the feasibility of the integrin α5β1 to be a specific marker of malignant transformation of the urothelial cells in humans and dogs. This study demonstrates that the integrin α5β1 is a marker of high-grade non-invasive UC, both in humans and dogs, and its expression is not mediated neither by pro-inflammatory environment nor by the process of benign hyperplasia, nor previous intravesical immune-/chemotherapy.

## Materials and methods

### Human patient cohort

The collection of clinical specimens was through transurethral resection of bladder tumor (TURBT) of the following cohorts: i) four non-oncological bladder samples from the follow-up of four prostate cancer patients after radical prostatectomy and radiotherapy, with bladder area characterized by normal urothelium or signs of actinic cystitis; ii) 10 low-grade and 10 intermediate-grade NMIBC from 20 patients; and iii) 43 high-grade neoplastic tissues from 27 bladder cancer patients (**Table 1**).

**Table 1:** Anamnestic data and tumor staging of human patients with high-grade urothelial carcinoma.

No.	Sex	Age	Smoke	Previous disease; year	Previous therapy	Current disease; year	α5 integrin expression
Bladder, non-tumoral tissue							
a	M	61	No	Prostate cancer; 2016	RRP, RT, hormone therapy	Actinic cystitis; 2022	0
b	M	80	No	Prostate cancer; 2011	RRP, RT	Actinic cystitis; 2021	0
c	M	69	No	Prostate cancer; 2010	RRP, RT	Actinic cystitis; 2019	0
b	M	70	ex	Prostate cancer; 2001	RRP, RT, Enantone + Casodex	Actinic cystitis; 2021	0
Bladder, *in situ* carcinoma (CIS)							
1	F	71	N.a.			CIS; 2023	0
2	M	74	Yes			CIS; 2023	0
3	M	69	No			CIS; 2023	0
4	M	62	Yes			CIS; 2022	0
5	M	74	Yes			CIS; 2023	1
6	M	61	No			CIS; 2022	1
7	M	90	No			CIS; 2023 CIS; 2023 CIS; 2023	1 1 1
8	M	65	Ex			CIS; 2023 CIS; 2023 CIS; 2023	1 1 1
9	M	77	No			CIS; 2023	0
10	F	80	No	pT1G3; 2021	BCG	CIS; 2022	1
11	M	69	Ex	pT1G3; 2020	BCG	CIS; 2022	1
12	M	53	No	CIS, pT1G3; 2017	BCG	CIS; 2022	1
13	M	73	Ex	CIS, pT1G3; 2018	BCG	CIS; 2022	1
14	M	70	Yes	CIS; 2022	BCG	CIS; 2023 CIS; 2023	1 1
15	M	81	No	pT1G3; 2019	BCG	CIS; 2023 CIS; 2023	1 1
16	M	78	Ex	pTaG1; 2018	MMC	CIS; 2023 CIS; 2023	0 1
17	M	74	No	CIS, pTAG3; 2022	BCG	CIS; 2023 CIS; 2023	0 1
Bladder, pTAG3							
18	M	74	Ex			pTaG3; 2022	1
19	F	78	Ex			pTaG3; 2022	1
20	M	78	Ex			pTaG3; 2023	1
21	M	68	Ex			pTaG3; 2023	1
22	F	75	Ex	pTaG3, CIS; 2022	BCG	pTaG3; 2023 pTaG3; 2023	0 1
23	M	85	No	pTaG3; 2014	BCG, MMC	pTaG3; 2022	1
24	F	79	Ex	pTaG2, CIS; 2021	BCG	pTaG3; 2023 pTaG3; 2023	1 1
14	M	81	Yes	pT1G3; 2019	BCG	pTaG3; 2023	1
17	M	72	No	pTaG3, CIS; 2022	BCG	pTaG3; 2022	1
Bladder, pT1G3 (integrin expression in luminal, infiltrating mass)							
25	M	69	No			pT1G3; 2022	1 (luminal) 0 (infiltrating)
26	M	85	No			pT1G3; 2022	1 (luminal) 0 (infiltrating)
6	M	61	No			pT1G3; 2022	1 (luminal) 0 (infiltrating)
21	M	68	Ex			pT1G3; 2023	1 (luminal) 0 (infiltrating)
27	M	72	Ex	pTaG1; 2002	MMC. Pharmorubicin	pT1G3; 2022	1 (luminal) 0 (infiltrating)
23	M	85	No	pTaG3; 2014	BCG, MMC	pT1G3; 2022 pT1G3; 2022	1 (luminal) 0 (infiltrating)

Four non-neoplastic tissues from four patients with actinic cystitis and 43 neoplastic tissues from 27 bladder cancer patients were collected by TURBT. A total of 25 CIS were diagnosed in 17 patients, 11 pTaG3 were diagnosed in nine patients, and seven pT1G3 were diagnosed in six patients. Five patients were diagnosed with high-grade tumors at different stages (#6, CIS and T1; #14 and #17 with CIS and Ta; #21 and #23 with Ta and T1). RRP, radical retropubic prostatectomy; RT, pelvic radiotherapy; BCG, *Bacillus* Calmette–Guerin; MMC, mitomycin C; N.a., not available. 0=negative; 1=positive.

All surgical specimens span the entire thickness of the bladder wall and were staged by experienced pathologists (RL, MC, and FS) according to the TNM classification (21) and morpho-architectural criteria according to the WHO classification (22). Formalin-fixed paraffin-embedded blocks were retrieved and stained using an automatic hematoxylin/eosin (HE) slide stainer (HistoCore SPECTRA ST, Leica). An expert genitourinary pathologist (R.L.) reviewed the HE slides.

*Canine patient cohort.* Canine clinical specimens were collected through TURBT from 12 treatment-naive dogs (23) (**Table 2**). Canine specimens were classified according to the most recent diagnostic criteria, and malignant tumors were graded according to the latest published grading system for canine urothelial carcinoma (24). The diagnosis of canine specimens was further confirmed according to the TNM classification (21) and morpho-architectural criteria according to the WHO classification (25) used for human specimens.

**Table 2:** Anamnestic data and tumor staging of dog patients with urothelial carcinoma.

No.	Breed	Sex	Age (years)	Anamnesis	Stage
1	American Staffordshire	Fs	8	N.a.	Normal urothelium
2	N.a.	N.a.	N.a.	N.a.	Benign hyperplasia
3	Border Collie	M	8	Urolithiasis	Cystitis
4	Beagle	Mc	8	Stranguria, polyps	Hyperplastic cystitis
5	Cocker Spaniel	Mc	12	Intravesical mass	Polypoid cystitis
6	Australian Sheperd	F	11	Intravesical mass	Non-infiltrating papillae (Ta)
7	Fox terrier	Fs	12	Hematuria	Non-infiltrating papillae (Ta)
8	Half-breed	Fs	11	N.a.	Invasive papillary UC (T1)
9	Jack Russel	Fs	15	Hematuria, pollakiuria, polydipsia	Infiltrating UC (T1)
10	Half-breed	Fs	11	Urinary tenesmus	Infiltrating UC (MIBC)
11	N.a.	Mc	12	Hematuria	Infiltrating UC (MIBC)
12	Half-breed	Fs	9	Intravesical mass	Infiltrating UC (MIBC)

Fs, female sterilized; Mc, male castrated; N.a., not available; UC, urothelial carcinoma.

### Immunohistochemistry analysis

Immunohistochemistry (IHC) on human and canine formalin-fixed paraffin-embedded tissues was conducted on 2-µm tissue sections that were deparaffinized with xylol and decreasing scale of ethanol. Antigen retrieval was performed with heat with Tris-EDTA buffer for 40 min at 97°C. After washing with TBS and quenching the endogenous peroxidase (5 min incubation with 3% H_2_O_2_), tissue sections were incubated for 10 min with 3%BSA/TBS to perform non-specific antigen blocking.

IHC for the alpha5 integrin was carried out with 1/400 dilution of rabbit recombinant monoclonal integrin alpha 5 antibody (clone EPR7854, Abcam) in Da Vinci diluent (Biocare Medical, Pacheco, CA, USA) for 1 h at room temperature. After washing, the binding of rabbit primary antibodies was detected using a Universal HRP-Polymer Biotin-free detection system (MACH4, BioCare Medical, USA) and 3,3-diaminobenzidine free base (DAB) as a chromogen. Tissue samples were then counterstained with Harris hematoxylin.

IHC for the beta1 integrin was carried out as reported above by incubating tissue sections with ½,000 dilution of rabbit recombinant monoclonal antibody (clone EPR16895, Abcam, Cambridge, CB2 0AX, UK). Negative controls for IHC analysis on both human and canine specimens were by omission of the primary Ab.

## Results

### The α5β1 integrin is expressed by neoplastic cells in high-grade human NMIBC

We previously reported that the α5β1 integrin is expressed in six out of eight (75%) patient samples diagnosed with carcinoma *in situ* (CIS) and by the orthotopic tumor in the MB49-derived pre-clinical murine model (19). In this study, we implemented this information by carrying out the analysis of an extended number of CIS and included specimens from high-grade pTa and pT1, for a total of 43 specimens of high-grade NMIBC and four non-tumoral bladders (**Table 1**). We also extended the study to low- and intermediate-grade NMIBC, pTaG1 (n=10), and pTaG2 (n=10), respectively.

**Figure 1:**
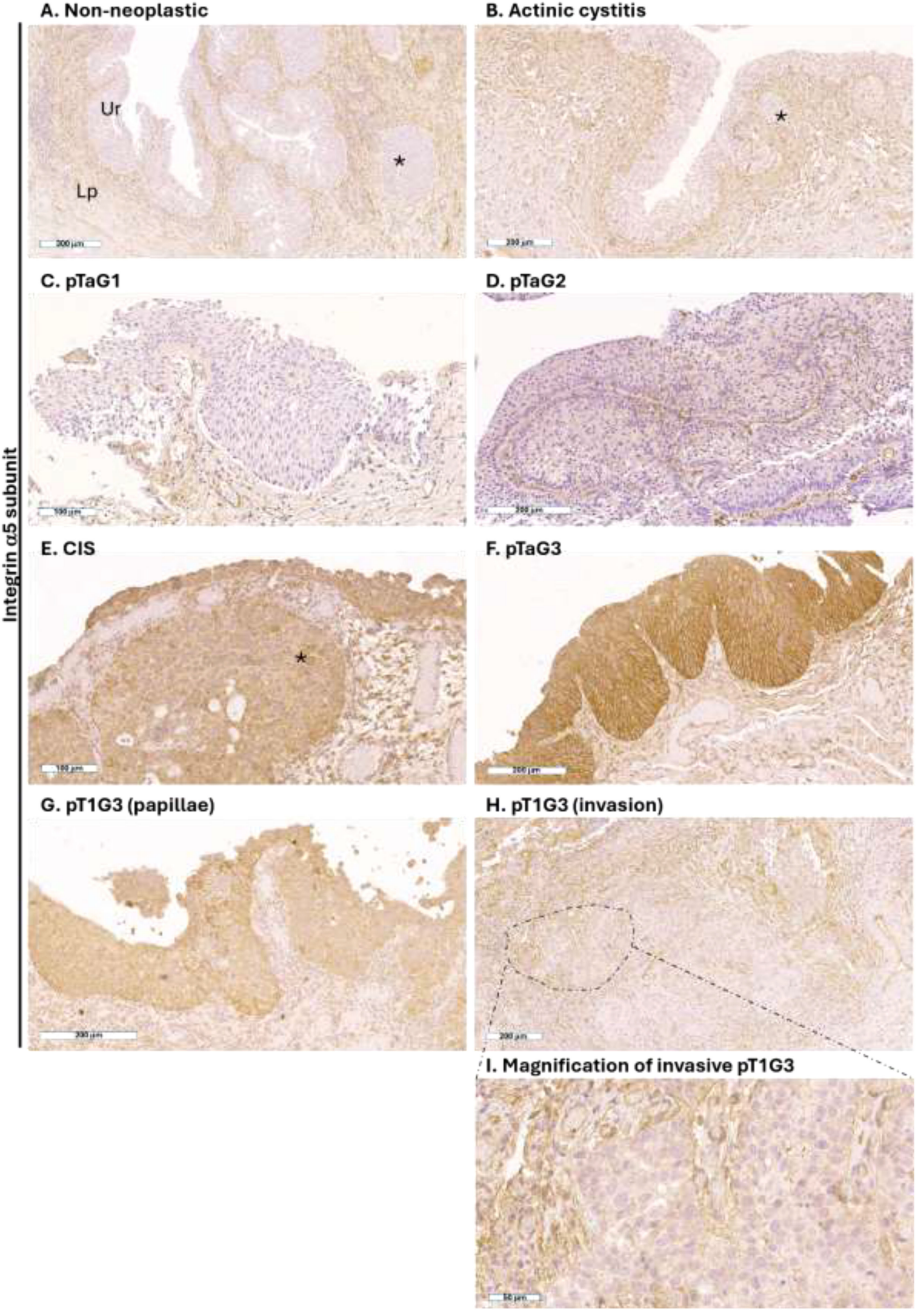
Expression of the α5 integrin subunit in human high-grade NMIBC. Representative immunohistochemistry photomicrographs of human bladder sections of non-neoplastic tissue **(A, B)**, pTaG1 **(C)**, pTaG2 **(D)**, CIS **(E)**, pTaG3 **(F)**, and pT1G3 in the urothelial layer **(G)** and in the lamina propria **(H)**; representative tumor area highlighted by dashed circle, and magnification in panel **(I)**; all tissues were obtained by TURB. Scale bar is reported at the bottom left of each panel. Ur, urothelium; Lp, lamina propria. *Representative Von Brunn’s nest.

In the non-tumoral tissues, the α5 subunit was expressed by the stromal cells present in the lamina propria but not expressed by the urothelial and umbrella cells present in the urothelium (**Figure 1A**). The same expression pattern was observed in the clinical specimen of chronic reactive urothelium following radiotherapy, characterized by irregular polarization of the nuclei of urothelial cells, hyperplastic umbrella cells, and inflammation in the lamina propria. In the same non-neoplastic tissues, also the cells in the Von Brunn’s nest were negative for the integrin α5 (**Figure 1B**).

In low-grade NMIBC (10 pTAg1), urothelial cells were negative for α5 subunit expression, as in non-tumor tissues. (**Figure 1C**).

In intermediate-grade NMIBC, membranous expression of the α5 subunit was identified on a minority of urothelial cells in pTaG2 of 4 out of 10 patients (**Figure 1D**), and no expression was identified in the urothelial cells in pTaG2 of six patients.

In the high-grade NMIBC specimens, the membranous expression of the α5 subunit was identified in the cancer cells of CIS (n=18/25 cases, 72%), including CIS that colonized the Von Brunn’s nest (**Figure 1E**), and high-grade pTa (n=10/11 cases, 91%) (**Figure 1F**). In high-grade pT1, the α5 subunit was expressed only in the non-invasive region of the tumor (**Figure 1G**); when tumor cells invaded the lamina propria (n=7/7, 100%), the α5 subunit was not expressed (**Figure 1H**), while the stromal cells surrounding the tumor cells expressed the integrin (**Figure 1I**).

**Figure 2:**
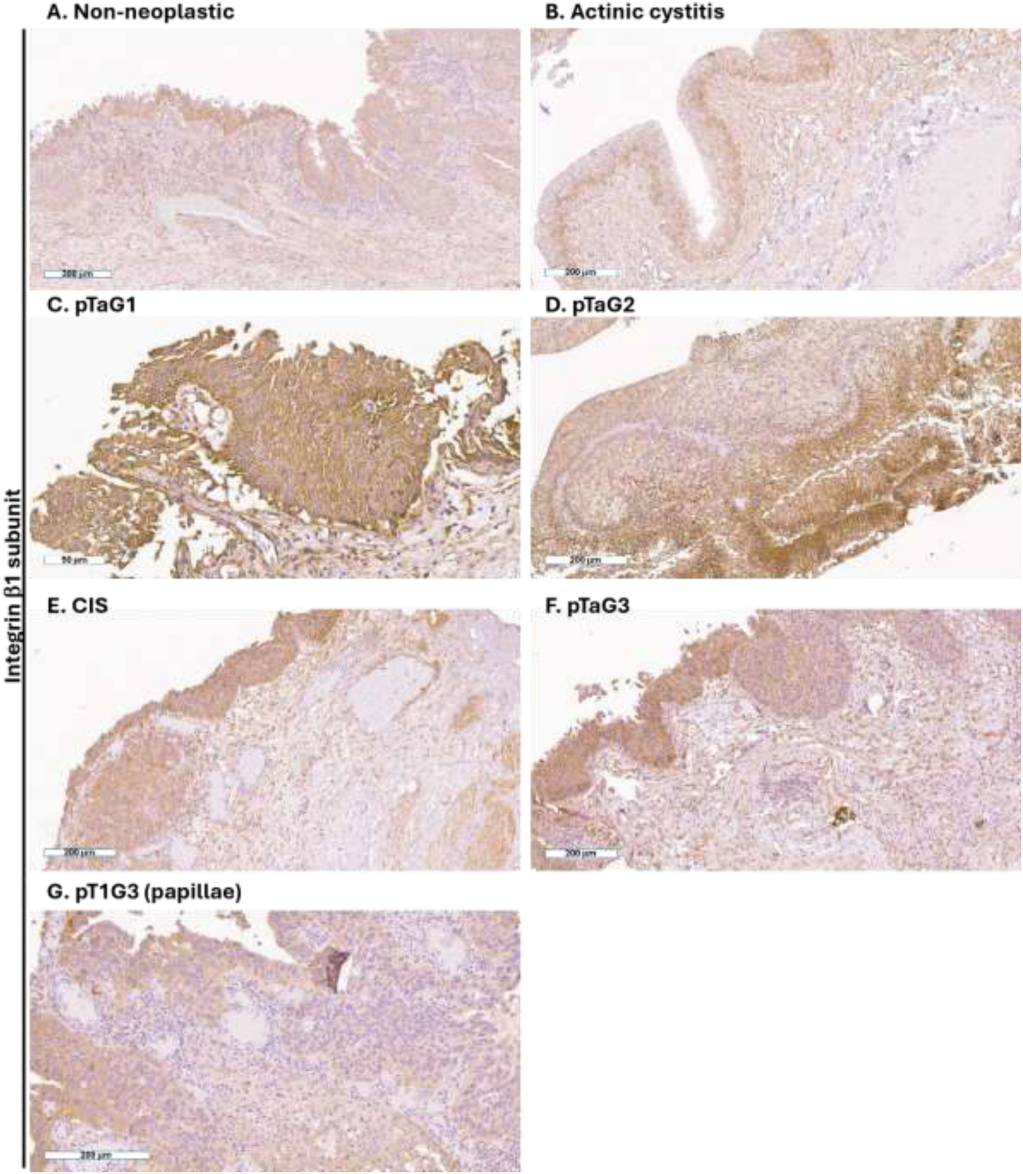
Expression of the β1 integrin subunit in human high-grade NMIBC. Representative immunohistochemistry photomicrographs of human bladder sections of non-neoplastic tissue **(A, B)**, pTaG1 **(C)**, pTaG2 **(D)**, CIS **(E)**, pTaG3 **(F)**, and pT1G3 in the urothelial layer **(G)**; all tissues were obtained by TURB. Scale bar is reported at the bottom left of each panel.

Integrin β1 subunit was always expressed by urothelial cells, such as in non-neoplastic condition (**Figure 2A**) that also includes chronic reaction as in the actinic cystitis (**Figure 2B**), low-grade UC (**Figure 2C**), intermediate-grade UC (**Figure 2D**), and high-grade UCs (**Figures 2E–G**). This result agrees with previous reports showing that integrin beta1 is expressed in all layers of the urothelium at intercellular junctions (26–28).

The outcome of this analysis shows that non-neoplastic urothelial cells and low/intermediate NMIBC were negative for the expression of the α5β1 integrin and that 81% (35 out of 43 cases) of high-grade NMIBC in the urothelial layer express the α5β1 integrin. Overall, these data suggest that the expression of the α5β1 integrin is specific to the neoplastic transformation of urothelial cells when high-grade neoplasia is in the urothelial layer but is not associated with chronic inflammation. Furthermore, the expression of α5β1 integrin by neoplastic urothelial cells was independent of previous intravesical treatment (**Table 1**), either immunotherapy with the BCG or chemotherapy with pharmorubicin or mitomycin-C.

### Integrin α5b1 expressed by high-grade canine UC

**Figure 3:**
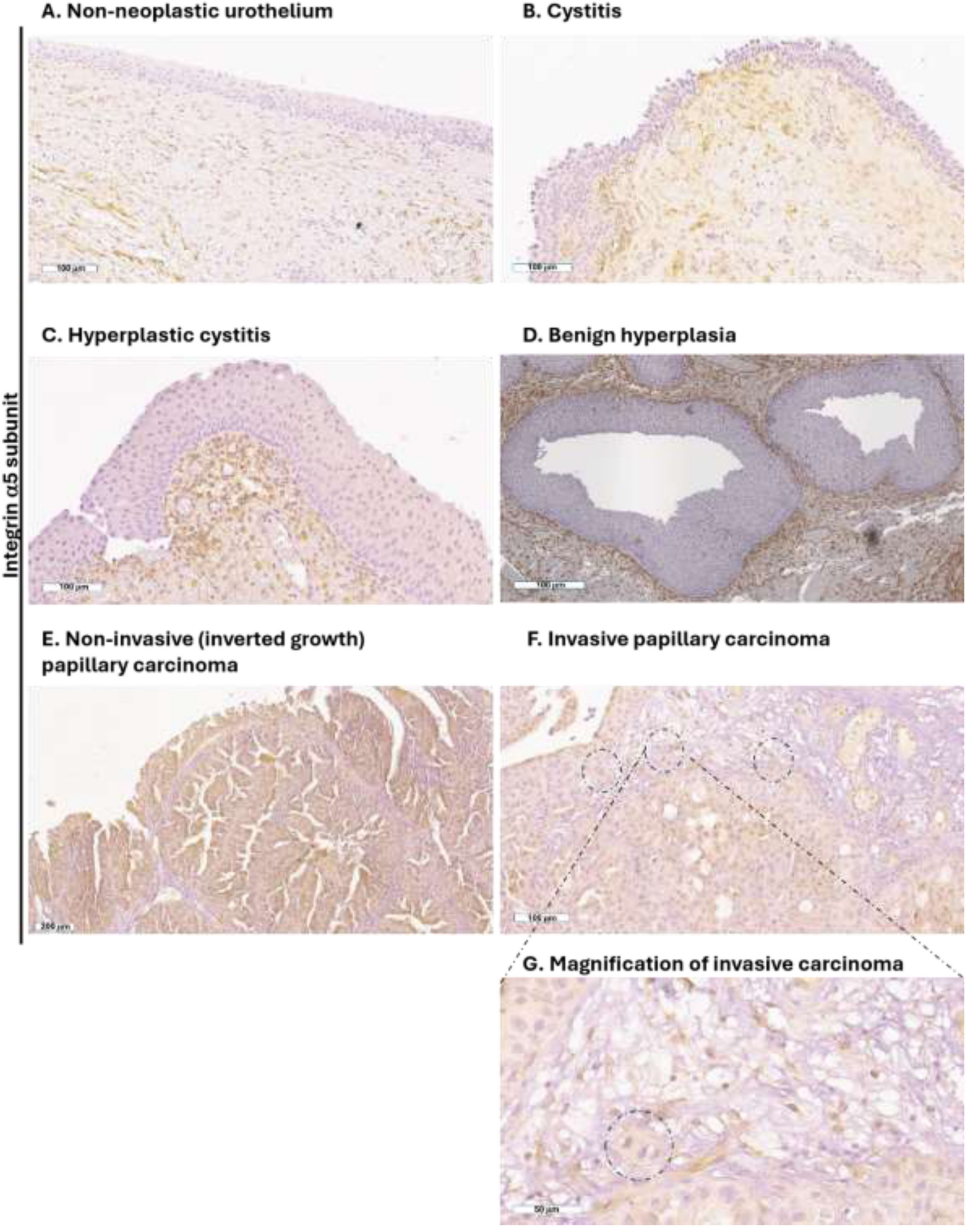
Expression of the α5 integrin subunit in canine UC. Representative immunohistochemistry photomicrographs of canine bladder sections of non-neoplastic tissue **(A-D)**, non-invasive papillary UC **(E)**, and invasive papillary UC spreading in the lamina propria **(F)**; representative tumor area highlighted by dashed circle, and magnification in panel **(G)**. All tissues were obtained by TURBT. Scale bar is reported at the bottom left of each panel.

Bladder specimens from dogs were validated by an experienced uro-pathologist (RL). Immunohistochemical analysis was carried out for the expression of the α5 and β1 chains of the integrin. In the non-tumoral tissues (specimens 1–5 in **Table 2**), the α5 subunit was expressed by the stromal cells present in the lamina propria but not expressed by the urothelial cells (**Figure 3A**). The same expression pattern was observed in the clinical specimen characterized by cystitis (**Figures 3B,C**), and benign hyperplasia (**Figure 3D**). In the canine UC, the α5 subunit was expressed also by the cancer cells in the non-invasive papillary carcinoma (**Figure 3E**) (specimens 6–8 in **Table 2**). Cancer cells that invaded the lamina propria were negative for the expression of the α5 subunit (**Figures 3F,G**) (specimens 9–12 in **Table 2**).

**Figure 4:**
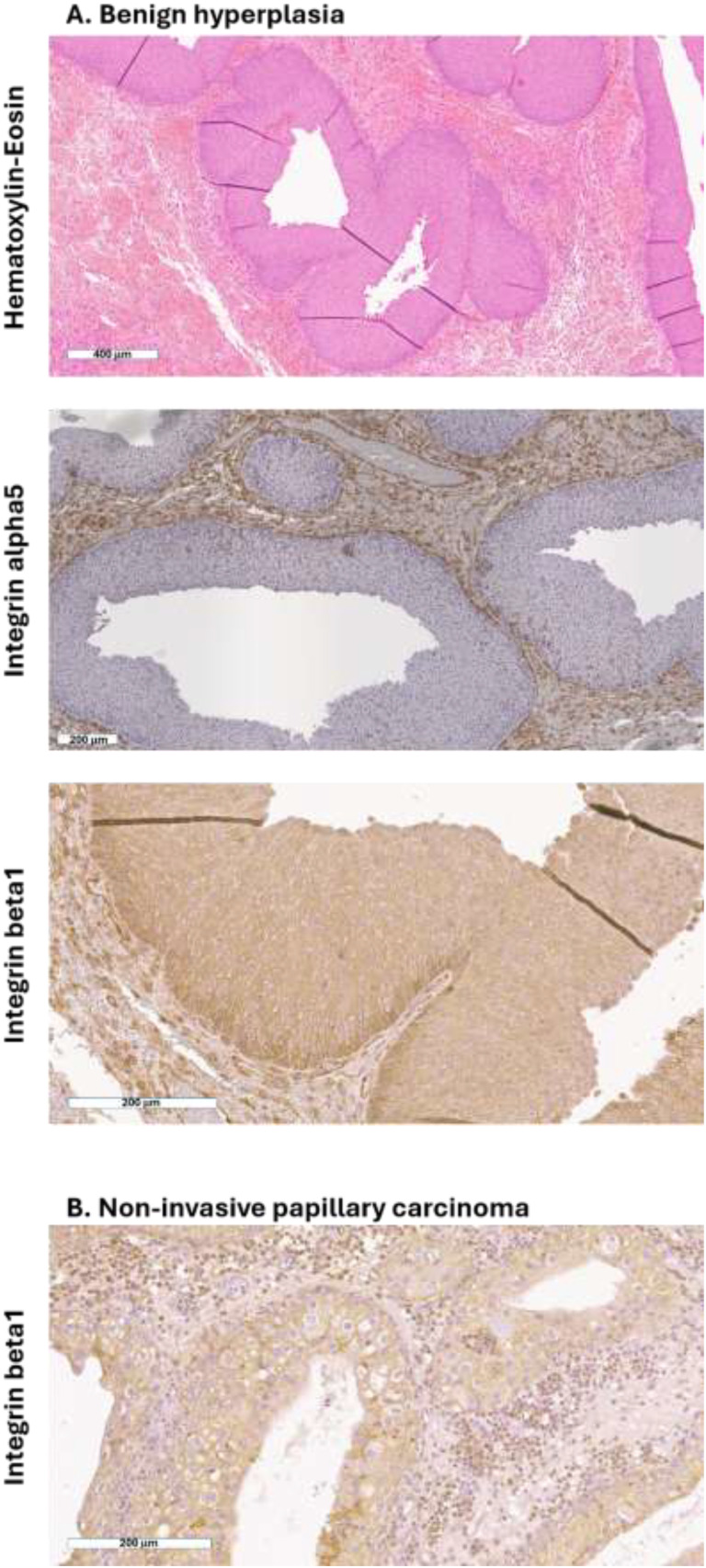
Expression of β1 integrin by normal and neoplastic canine urothelial cells. Representative photomicrographs of **(A)** hematoxylin–eosin and immunohistochemistry for α5 and β1 integrins of canine bladder section with benign hyperplasia and **(B)** immunohistochemistry for β1 integrin of canine bladder section with non-invasive papillary carcinoma. Scale bar is reported at the bottom left of each panel.

As recently reported in human and murine bladder section (19), we here confirmed that the β1 subunit was expressed by normal urothelial, stromal, and tumor cells of the canine bladder. The β1 subunit was expressed by normal urothelial and stromal cells, and the same expression was observed for benign and malignant neoplasia (**Figure 4**).

## Discussion

Outcomes from this study show that the integrin α5β1 is a marker of high-grade non-invasive UC, both in humans and dogs. The integrin α5β1 is specifically expressed by the high-grade neoplastic urothelial cells, and its expression is not mediated neither by pro-inflammatory environment nor by the process of benign hyperplasia, nor by previous intravesical immune-/chemotherapy. We have recently reported that the integrin α5β1 is also expressed by the orthotopic bladder cancer in the MB49 murine model but not by the normal murine epithelium (19). The second outcome of this study is that the specific expression of the integrin α5β1 by high-grade bladder cancer cells is conserved among the mouse, dogs, and human species.

The integrin α5β1 was reported to be expressed also in other neoplasia of epithelial origin such as ovarian and cervical tumors in which 80% and 84% of tumors are positive for the expression of integrin α5β1 and significantly correlate with higher clinical stage (29, 30). We here report that 81% of the high-grade NMIBC express the integrin α5β1 but only when the neoplastic cells are in the luminal area, while neoplastic cells in the lamina propria (T1 stage) do not express the integrin α5β1. The expression of the integrin α5β1 by the malignant cells is likely to represent one of the steps of the tumorigenesis of epithelial tumors. It has been reported that silencing of α5 integrin in tumor cells strongly reduces lung tumor spreading (31). Concerning the mechanism, it has been previously reported that α5β1 and αVβ3 integrins directly activate matrix metalloproteinases and thus participate in the degradation of the components of the extracellular matrix of the basement membrane to support tumor invasion (32, 33). Once in the lamina propria, the lost expression of the integrin α5β1 could be part of a process to facilitate cancer cell movement, while degradation of the basement membrane of the endothelium by the integrin αVβ3 supports intravasation and spreading of the disease (34). Overall, the expression of the integrin α5β1 by the high-grade malignant cells is likely to represent one of the steps in the progression of epithelial tumors (35).

The identification of the integrin α5β1 expressed by high-grade bladder cancer cells located in the urothelium can be used for the delivery of contrast agents aimed to carry out targeted diagnostic imaging solutions in either therapy-naive patients or patients with relapsing high-grade UC. Not being expressed by the urothelial cells in the conditions of hyperplasia, cystitis, and chronic inflammation, but being specific for the high-grade cancer cells, the targeting of integrin α5β1 could overcome the limitation of white and blue light cystoscopies that detects tumor and areas of inflammation that arise following TURBT or BCG instillation (36–38).

The integrin α5β1 can represent a target that can be exploited using molecules recognizing the heterodimer, such as cyclic peptide (19, 20), peptidometic ligand (39) or antibody (40), or the integrin α5 subunit through an aptamer (41). Depending on the resolution of the diagnostic imaging techniques, lesions of different sizes can be diagnosed. Peptidomimetic ligand that delivers ^68^gadolinium was developed as a contrast agent for PET imaging (39). The application of engineered gold nanorods targeting the integrin α5β1 as the contrast agent in photoacoustic imaging has recently demonstrated to diagnose bladder cancer lesions <1mm in the preclinical murine model (19, 20, 41). Deployment of this solution in the clinical scenario could offer a novel strategy that complements the TURBT approach, which allows for more accurate detection of the disease and identification of the residual disease in patients undergoing i) second-look TURBT and ii) enrolled in bladder-sparing protocols (42). Likewise, the integrin α5β1 also represents a promising therapeutic target for the gold nanorods-assisted hyperthermia of orthotopic bladder cancer (20).

The integrin α5β1 can be used to deliver a targeted contrast agent for diagnosis or therapy against high-grade UC both in humans and dogs and to reduce the high tumor recurrence rate observed in both species. Dogs and humans UC share many characteristics, but the shorter lifespan of dogs mean that tumors develop, metastasize, and respond to therapy more quickly (3). Given the close similarity between high-grade UC in humans and dogs, but the shorter lifespan of dogs, the spontaneous high-grade UC in dogs can be a helpful model to speed up the transition of drugs tested in murine models in clinical trials, with results that can impact both humans and dogs.

## Data Availability

The original contributions presented in the study are included in the article/supplementary material. Further inquiries can be directed to the corresponding author.
