# Correction: High-grade non-muscle invasive urothelial carcinoma in dogs and humans share specific expression of integrin α5β1

**DOI:** 10.3389/fonc.2025.1640082

**Published:** 2025-06-25

**Authors:** Roberta Lucianò, Maurizio Colecchia, Francesca Sanvito, Irene Locatelli, Chiara Venegoni, Alessia Di Coste, Davide Danilo Zani, Angelica Stranieri, Chiara Giudice, Antonella Rigillo, Matteo Gambini, Francesco Montorsi, Andrea Salonia, Marco Moschini, Massimo Alfano

**Affiliations:** ^1^ Department of Pathology, Istituto di Ricovero e Cura a Caratterle Scientifico (IRCCS) San Raffaele Scientific Institute, Milan, Italy; ^2^ Università Vita-Salute San Raffaele, Milan, Italy; ^3^ Division of Experimental Oncology/Unit of Urology, Urological Research Institute (URI), Istituto di Ricovero e Cura a Caratterle Scientifico (IRCCS) San Raffaele Scientific Institute, Milan, Italy; ^4^ Department of Veterinary Medicine and Animal Science, University of Milan, Lodi, Italy; ^5^ I-Vet Diagnostica Veterinaria, Brescia, Italy

**Keywords:** urothelial cancer, human, dog, integrin, marker, bladder

In the published article, there was an error in the **Funding** statement. “The author(s) declare that financial support was received for the research and/or publication of this article. This work was supported by funding of the Italian Ministry of Health [Ricerca corrente].” The correct **Funding** statement appears below.

“The author(s) declare that financial support was received for the research and/or publication of this article. This study was funded by the European Union’s Horizon EUROPE; PHIRE project is funded under grant agreement no. 101113193 (https://cordis.europa.eu/project/id/101113193). The funding source had no role in the design of this study, data interpretation, or writing of the manuscript. Views and opinions expressed are those of the author(s) and do not necessarily reflect those of the EU or the EIC.”

The original version of this article has been updated.

